# Consumer Response to Novel Foods: A Review of Behavioral Barriers and Drivers

**DOI:** 10.3390/foods13132051

**Published:** 2024-06-27

**Authors:** Cihat Günden, Pelin Atakan, Murat Yercan, Konstadinos Mattas, Marija Knez

**Affiliations:** 1 Department of Agricultural Economics, Ege University, 35040 İzmir, Turkey; cihat.gunden@ege.edu.tr (C.G.); murat.yercan@ege.edu.tr (M.Y.); 2 Department of Agricultural Economics and Agribusiness, Yaşar University, 35100 İzmir, Turkey; 3 Department of Agricultural Economics, Aristotle University of Thessaloniki, 54124 Thessaloniki, Greece; mattas@auth.gr; 4 Capacity Development Network in Nutrition in Central and Eastern Europe (CAPNUTRA), 11000 Belgrade, Serbia; marijaknez186@gmail.com; 5 Centre of Research Excellence in Nutrition and Metabolism, Institute for Medical Research, University of Belgrade, 11060 Belgrade, Serbia

**Keywords:** novel food, consumer response, behavioral barriers, food innovation

## Abstract

There is a pressing need for a transition toward more sustainable diets, which has become a shared priority for both consumers and businesses. Innovation is becoming increasingly widespread across all facets of the food supply chain. This innovation spans various domains related to production, including sustainable cultivation methods as well as new food technologies like gene editing, new product development like functional foods, and revitalizing underutilized and genetically diverse varieties to preserve biodiversity. However, not all innovative efforts are accepted by consumers and survive in markets. The interwoven and long agri-food supply chains often obscure the feedback loop between production and consumption. Consequently, it is important to understand to what extent consumers embrace these food innovations and form new eating habits. This review aims to investigate the consumer response to novel foods, focusing on behavioral factors, which have yet to receive as much attention as sensory factors. Peer-reviewed empirical articles from the last decade are examined inductively to develop a bird’s-eye view of the behavioral barriers to and drivers of consumer acceptance of novel foods. In addition, strategies to overcome the identified challenges associated with the behavioral barriers are reviewed and examined. Based on this, the study links cognitive biases with behavioral factors influencing consumer acceptance of novel foods. This study concludes that the inconvenience associated with abandoning established eating habits is typically perceived as a loss, and avoiding this inconvenience is deemed more worth the risk than the potential gains associated with novel food consumption. This study suggests that framing and placing pro-diversity labels could serve as effective behavioral interventions for marketing strategists and food policymakers.

## 1. Introduction

As the world’s population has grown and agricultural production patterns have shifted toward resource-intensive monoculture, the strain on the environment has become increasingly evident [1]. In addition to monocropping, the overreliance on a limited number of crop species and protein sources further exacerbates biodiversity loss and contributes to environmental degradation [2,3]. The link between dietary patterns and biodiversity is increasingly recognized, highlighting the urgent need to transition toward more sustainable diets that prioritize agrobiodiversity, alternative food sources, and resource efficiency in food production [2,3,4,5].

Embracing more sustainable diets is a common concern for both consumers and businesses. However, the concept of “sustainability” can vary in interpretation and often lacks biodiversity as a goal [3,5]. One prevalent trend involves transitioning to diets with reduced reliance on animal products, exemplified by the Mediterranean diet in Europe. The Mediterranean diet (MD), in comparison with other prevalent European dietary patterns, exerts less pressure on biodiversity [4] and is beneficial for human health [6]. The challenge when adopting the MD is not about crop availability but about crop utilization in the European case [7]. A complementary action to adopting the MD could involve revitalizing forgotten, underutilized, and genetically diverse species. Gaining increasing consumer attention because of their richness in micronutrients, vitamins, and proteins, “Neglected and Underutilized Species” (NUS) are agricultural species that were previously cultivated but are no longer cultivated in significant quantities due to economic, cultural, agronomic, or genetic factors [8,9,10]. The term can refer to varieties, species, or crops, often indicating that they have been overlooked by researchers, breeders, or policymakers [10]. Some underutilized crops are still cultivated and consumed as minor crops but not traded [11]. In addition to providing benefits to human health and nutrition, these crops also play a role in conserving biodiversity [11]. This emphasis on diverse and nutrient-rich food sources ties into broader efforts to address challenges within the food system.

On the other hand, often coupled with sustainability concerns, innovative food technologies have driven the search for new food sources and production processes [12]. Commonly referred to as novel foods, newly developed foods are often the products of cellular agriculture, insect farming, algae cultivation, 3D-printed food, or extraction techniques [12,13]. A typical novel food usually focuses on one or more of the following benefits: exploitation of new resources with low environmental impact or by-products of another production process, cost minimization, production of food with a high level of nutritional value [12,14]. Depending on the relevant national regulation, what is considered a novel food varies [13]. The Novel Food Regulation enforced by the European Union (EU) defines novel food as newly developed, innovative food or food produced using new technologies and production processes, as well as food traditionally eaten outside of the EU [15]. This includes alternative protein sources for Western cultures, such as edible insects or algae, functional foods, value-added surplus products, and foods produced with new technologies such as cellular agriculture, gene editing, and indoor farming. According to the regulation, to qualify as novel food, the food must be safe for consumers and adequately labeled so as not to mislead consumers. Moreover, if novel food is intended to replace another food, it must not differ in a nutritionally disadvantageous way for consumers. The EC enforces several rules for the market authorization of novel foods to ensure food safety [16]. Similar to novel food regulations in different countries, the primary motivation behind the EU Novel Food Regulation is to maintain food safety rather than to directly reflecting global sustainability concerns [13].

Beyond safety concerns and market regulations, consumer acceptance is an important factor determining the market success of novel foods [12] and, eventually, realizing the promised or unwanted environmental impact associated with their production [5]. Consumers continuously make choices about what, when, where, with whom, and how they eat, thereby shaping their eating behavior [17]. Food choice is influenced by sensory, physiological, demographic, and socio-economic factors, as well as by perceptions, expectations, values, social influences, and habits [17,18]. These elements contribute to varying degrees to the formation of behavioral factors that are often very difficult for businesses to read for various reasons, including the intertwined and long agri-food supply chains [3]. This study aims to explore the body of literature on novel food, focusing on the behavioral factors that influence consumer choice. The behavioral barriers and drivers regarding consumer acceptance of novel food are examined using an inductive approach. Then, strategies to eliminate or minimize behavioral barriers are outlined. In the next section, the behavioral factors that influence consumers’ food choices are elaborated in the context of new and unknown foods. The subsequent section informs about the methodology employed in this review. The following section elaborates on the most studied factors based on the outcomes of the query. Subsequently, a compilation of strategies to enhance the acceptance of novel food is presented. Finally, the concluding remarks encompass this study’s limitations and prospective avenues for future research endeavors. This study is part of the BioValue Project, an EU-funded project within the framework of Horizon 2020. The project’s goal is to encourage the incorporation of NUS into food value chains to improve biodiversity. NUS potentially fall under the EU Novel Food Regulation’s category of “traditional food eaten outside of the EU”; however, European agriculture also has its own NUS, which could easily reappear in dietary habits [19]. In other words, given the health and biodiversity benefits, encouraging the broader consumption of NUS can be as promising as other novel foods. In this direction, the present study intends to examine the consumer response to NUS as well as to novel foods as defined by the EU Regulation.

## 2. Consumer Response to Novel Foods and Behavioral Factors

Consumers’ willingness to experiment with novel food and repeat their consumption depends on various factors. Taste, appearance, flavor, odor, and texture are among the properties that co-construct a product’s sensory properties [20,21]. Their role in the acceptance of novel food is undeniable, yet it is not sufficient. The combination of psychological, social, and cultural factors influences how consumers perceive, process, and respond to information about food and, ultimately, their food purchase and eating behavior. People often make consumption choices as an expression of their own values, and this remains true when it comes to food choices [22]. When consumers find certain attributes of a food relevant, they may be more motivated to consume it. The environmental benefits of cultured meat [23] or the health claims associated with functional snacks [24] may play a role in the acceptance of novel foods. In addition to values, personality traits are also closely related to food choice. Many studies suggest that personality traits such as openness to new experiences, extraversion or agreeableness may be associated with a higher rate of experimenting with novel food [25,26]. On the other hand, individuals often follow social norms regarding food choices because norms contain critical information about the safety, nutrition, or appropriateness of a particular food or way of eating [27]. In addition to norms, individuals typically respond to temptations, especially when social cues indicate appropriateness [28]. Moreover, most food choices are habitual behaviors, typically outside people’s conscious awareness and susceptible to manipulation by environmental cues [29]. Factors contributing to the formation of habitual eating behaviors are closely related to an important phenomenon known as familiarity.

Familiarization with food is a significant driver that builds expectations about food. Familiarization starts with early exposure during childhood, and there is a strong correlation between exposure to food and reinforcement of familiarity with it [30]. Familiarity with food develops certainty about expectations, and it often creates an advantage over unfamiliar or novel food products [21,30]. It may imply texture familiarity [21], taste familiarity [31] or cultural familiarity [32]. On the other hand, familiarity with the food also affects consumers’ food evaluation process. When evaluating familiar foods, consumers often rely on previous experience, so they find it sufficient to focus on just a few key characteristics. In this way, they can make quick, less cognitively demanding and precise judgements. However, the evaluation process for novel foods is different. Consumers are likely to evaluate an unfamiliar food more comprehensively during sensory testing, and it is often difficult to make a distinct like or dislike judgement [33,34].

Unfamiliarity with a food often leads to “food neophobia”, which is “the reluctance to eat and/or avoidance of novel foods” [35]. Demonstrating food neophobia is an evolutionary adaptation of humans (along with many omnivores) because consuming familiar foods is a strategy for avoiding dangerous foods [36]. It has attracted considerable attention from researchers as one of the major psychological barriers to the acceptance of novel food [30,36].

**Figure 1:**
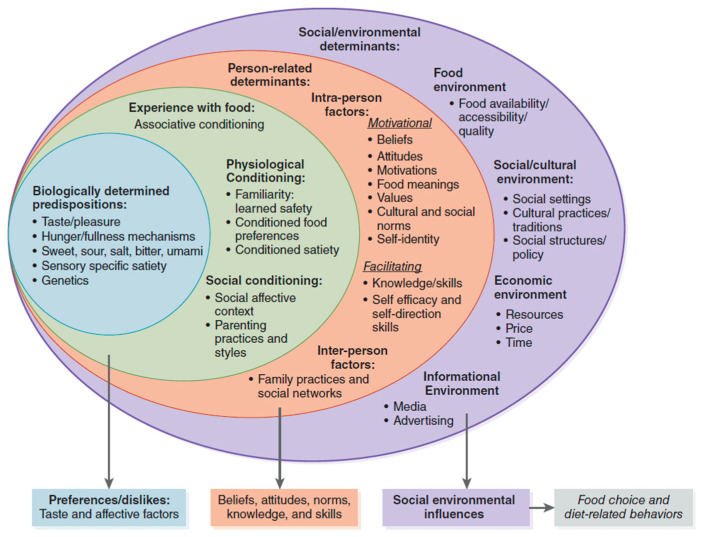
Factors influencing food choices and diet-related behaviors [37].

The consumer response to novel foods can be interpreted as the collective result of various interconnected factors, including those briefly illustrated above. Contento’s Framework allows for a broader perspective by focusing on the interrelationships among the many factors that contribute to the formation of food choice and behavior (Figure 1). The framework categorizes the elements contributing to food choice and dietary behavior arising from biological, experiential, personal, social, and environmental factors. All the factors are interrelated, and larger circles accommodate the influence of smaller circles [37].

Eating behavior is often related to what individuals attach value to, and it is strongly influenced by global megatrends, among other factors. In 2014, Mehmeti et al. identified four megatrends that will impact new food development over the next ten years: health and wellness, convenience, pleasure, and natural and organic foods in response to consumers’ sustainability values [38]. Accordingly, in 2020, Boukid suggested that two critical trends expressed as “better for you” and “better for the planet” influence the intention to consume plant-based meat analogues, which have become more mainstream since 2015 [39]. Health concerns have become even more pronounced following the COVID-19 pandemic [40,41], possibly accelerating the spread of the impact of these two megatrends more broadly, both regionally and intergenerationally.

## 3. Methodology

**Figure 2:**
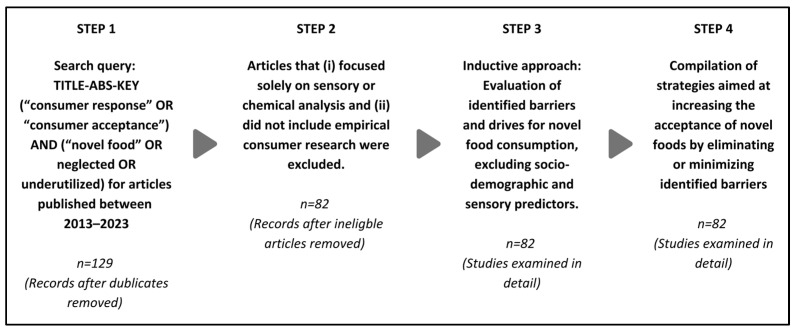
Methodology.

To obtain a bird’s-eye view of existing studies on the consumer response to novel foods, the Web of Science and Scopus databases were used, as both allow advanced searches on specific topics, stipulate certain quality criteria, and include only peer-reviewed journals [42]. To screen relevant articles, the keyword “novel food” as an umbrella term that naturally denotes the innovative aspect of new foods and carries unanswered questions about consumer choice and market success was used to narrow the results. The keywords “underutilized” and “neglected” were added to broaden the search, as these categories are not usually referred to as novel food but are essential for this review study due to their potential contribution to biodiversity efforts and the fact that aspects of consumer acceptance of these foods remain largely unexplored [11]. The keywords “consumer response” and “consumer acceptance” were included in the query to direct our attention to the articles focusing on the consumer response to novel foods. The articles published between 2013 and 2023 were screened using the query TITLE-ABS-KEY (“consumer response” OR “consumer acceptance”) AND (“novel food” OR neglected OR underutilized). The query resulted in 129 articles after removing duplicates. In the next step, articles that (i) focused solely on sensory or chemical analysis and (ii) did not include empirical consumer behavior research were excluded. The remaining 82 articles were included in this study and evaluated thoroughly. Employing an inductive approach, factors contributing to the formation of behavioral barriers and drivers concerning novel food consumption were reviewed and further evaluated. Following this, strategies to handle obstacles to consumer acceptance of novel food were examined (Figure 2).

## 4. Results 

The primary purpose of this review was to explore the body of literature on novel food, focusing on the behavioral factors that influence consumer choice. This section reports the findings of a thorough review of 82 articles that contain empirical results on consumer acceptance of novel foods, which were selected based on the criteria described in the previous section. The following subsection describes the main attributes of the novel foods analyzed in these studies. Subsequent subsections are devoted to detailing each of the factors identified in these studies as impacting the consumer response to novel foods, ultimately becoming a behavioral barrier or driver. The final subsection reviews the strategies to overcome the behavioral barriers identified in these studies.

### 4.1. Main Attributes of Novel Foods

As a reflection of the increasing protein demand and growing environmental consequences of conventional livestock management practices, searching for ways to embed alternative protein sources into diets dominates consumer behavior research concerning novel food. Out of 82 studies, 52 focus on alternative protein sources such as cultured (lab-grown) meat, whole insects, insect-based products, insect-fed animal products, whole and processed worms, plant-based meat, spirulina-based products, algae, and surimi (see Appendix A Table A1). Among the alternative protein sources, edible insects have garnered the most attention due to their versatility as an ingredient in various processed foods, almost indistinct sensory attributes, and high protein content [43,44]. Nearly all the studies focusing on alternative protein addressed the increasing global population, protein demand, and environmental damage caused by widespread livestock production. Only a few mentioned ethical considerations related to animal welfare [45,46,47,48,49,50]. This was followed by concerns about malnutrition and health, which were predominantly mentioned by the studies on functional and biofortified foods [51,52,53]. The food systems’ circularity was the main motivation for value-added surplus products (VASPs) [54,55,56]. The studies focusing on novel food technologies such as gene editing, gene modification, or three-dimensional food printing did not address global food challenges, except for a few [57,58]. Only three studies focused on revitalizing underutilized (i.e., forgotten) foods: spirulina, breadfruit, and baobab [32,59,60]. These studies primarily focused on nutritional benefits, except for the one on spirulina, an alternative source of protein, which was also motivated by challenges related to the increasing protein demand [32]. In general, indoor farming and novel food technologies also garnered attention, often addressing concerns related to food security and safety [61,62,63,64,65]. Decreasing biodiversity and cultural diversity related to food production and concerns regarding social sustainability were not the primary motivations of any of the reviewed studies despite their direct relevance to food system sustainability [2].

### 4.2. The Influence of Familiarity

Familiarity’s direct or mediating influence on the consumer acceptance of novel food has mainly been studied [18,31,32,44,48,56,62,66,67,68,69,70,71,72,73,74,75,76]. Familiarity can act as a mediator for food choice [32,73] and can even mitigate the impact of disgust [71]. Familiarity with different cultures, exotic foods, or experimenting with new foods can influence novel food acceptance [56]. The influence of familiarity on consumer acceptance is usually significant. The level of familiarity is a decisive factor for consumer segmentation, often surpassing the power of socio-demographic factors [66,75]. Taking advantage of the established familiarity with a product by including novel ingredients in familiar foods is widely used as a strategy to increase consumer acceptance of novel products, especially if this new ingredient has previously been categorized as “inedible”. For example, studies that attempt to explore the acceptance of the consumption of insects or worms as alternative sources of protein claim that harmonizing them in familiar products can help, yet it is often not sufficient [31,32,43,44,70,71,74,77,78,79,80]. Familiarity can be built in various ways. For example, appropriate product design [31,43] or communicating the sustainability benefits or production methods through labels or written messages to consumers [71,81] can facilitate the process of acceptance. When these strategies meet consumers’ values and needs, the impact of familiarity increases [71]. This finding underlines the importance of consumer segmentation studies. Furthermore, positive sensory experience (taste, texture, etc.) also creates familiarity and therefore influences the maintenance of food intake [44,74,80,82]. 

### 4.3. The Role of Disgust and Food Neophobia

Disgust is one of the primary factors that can hinder the acceptance of novel food, especially if it contains edible insects, cultured meat, or worms [26,47,48,49,57,66,68,71,77,78,80,83,84,85,86,87]. Disgust often co-exists with the perceived risk related to food intake [26,69,77,84], cultural inappropriateness [68,69,77,84], perceived unnaturalness [48,49], and food neophobia toward entomophagy [47,80,86,88]. The effect of food neophobia and disgust has been found to be significant in many studies on entomophagy or alternative meat: it may even repress other personal traits or the motivation to eat more sustainably or healthily [78,80]. In line with the significance of the impact of disgust on consumer acceptance, Barbera et al. suggest that disgust can be used as a tool to segment consumers, as it usually does not coexists with certain personality traits, such as openness, regardless of socio-demographic differences in the highly representative sample in their study [26]. On the other hand, the significance of disgust has generally been reported to be lower for new plant-based novel foods, which are often more familiar to consumers [51]. The effect of food neophobia as a barrier to the consumption of novel food may escalate when the physical disadvantages of individuals increase [53]. On the other hand, its impact can decrease with positive experiences and repeated consumption [44,82] as well as through information provision about health, safety, or environmental claims [44,84].

### 4.4. Consumer Trust and Fear of Novel Food Technologies

Consumers commonly perceive novel food technologies with trust and fear. Novel food technologies such as gene editing, 3D-printing, cellular agriculture, nanotechnology, and the like are usually not familiar to most people, thus possibly prompting the urge to evaluate the safety of the food. Consumers may turn to trust in different sources. They may rely on their perception of the system that ensures safety standards [59,89,90] or the food technology used to produce the food [79]. The outcomes of individuals’ cognitive processes also contribute to the final decision to trust and consume products made by novel food technologies. Attitudes toward novel food technologies, shaped by beliefs, personal characteristics, and values, as well as by the sense of control over the technological processes in question, significantly influence acceptance [61,91].

When consumers associate fear rather than trust with novel food technologies, it establishes a solid psychological barrier known as Novel Food Technology Neophobia (NFTN) [79]. The perceived risks and uncertainties may increase due to unfamiliarity with new technologies, resulting in behavioral barriers to novel food consumption [73,91]. NFTN can be observed in conjunction with different concerns regarding the essence of the technology in question. A perceived lack of naturalness emerges as one of the most critical barriers to acceptance when it comes to novel food technologies, such as 3D printing [57,79], cellular agriculture [48,49], and indoor farming [65].

### 4.5. The Role of Motivations and Contextual Factors

Personality traits, values, and beliefs play a role in influencing the motivation to accept novel food. People with an interest, general openness, curiosity, or adventurousness show more interest in trying novel foods [26,46,48,68,76,78,83,85,92,93,94,95,96]. The benefits for environmental sustainability, health, and animal welfare typically motivate consumers toward alternative protein products [94,97,98]. However, higher motivations do not always translate into actual behavior, such as trying or continuing to consume the novel food [54,55,77]. Additional incentives are necessary to encourage the integration of novel foods into dietary habits beyond mere willingness to try them. The factors influencing repetitive consumption are practical and contextual, such as price, taste, availability, and “fit” with established eating practices [95]. In fact, price has been consistently found to be influential for actual novel food consumption behavior [50,54,66,73,76,97,99,100,101,102]. Different research has highlighted the significance of both availability and convenience in fostering the acceptance of novel foods [18,51,75,77,94,100,103].

The context and time affect the willingness to try novel food. The deficient availability of food in general (i.e., a survival context) can increase novel food consumption by overriding the factors that would hinder the acceptance of novel food under normal conditions [66,68]. On the other hand, socially motivating environments may boost the willingness to try novel foods. Studies show that when individuals associate the consumption of a novel food with a sense of togetherness or acceptance in a community, their willingness to try increases [18,77,104,105]. The reverse scenario may pose a barrier to consumption [69]. Examples include when individuals associate food with cultural meanings, such as it being perceived as food of the poor [59], incompatible with the local eating culture [77], a threat to masculinity [80], or contrary to religious and racial dietary customs [99].

### 4.6. Strategies to Improve the Acceptance of Novel Food

**Figure 3:**
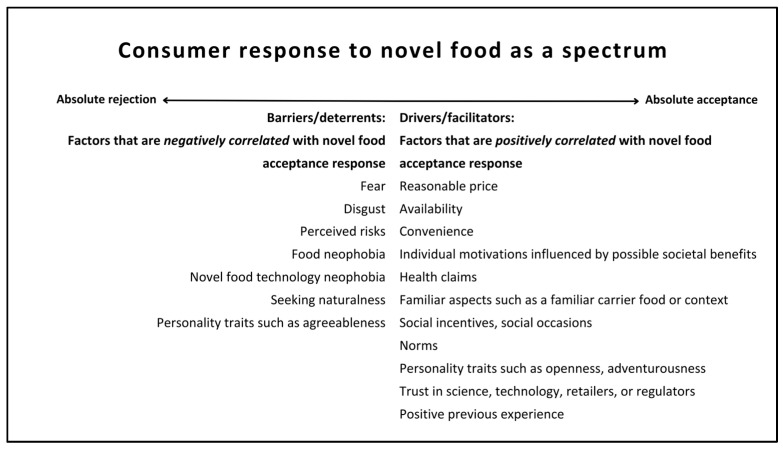
Consumer response to novel foods as a spectrum (the order of the factors does not imply magnitude) (source: authors, based on the results).

The results of this review suggest that a wide array of behavioral factors influences the consumer response to novel food. The individual impact of these factors on the consumer response remains in question, primarily due to the difficulty of evaluating many different factors simultaneously. The consumer response to novel food can be viewed as a spectrum. Each behavioral factor can move consumers across the spectrum to some extent, but none alone is enough to move them entirely in one direction (Figure 3). This suggests that the strategies to increase consumer acceptance of novel foods should include actions that address a combination of various behavioral barriers and leverage a combination of multiple behavioral drivers.

Providing information is one of the most cited strategies for increasing consumption of novel food. This tendency is often based on the assumption that consumer ignorance (or uncertainty) about novel food is the most crucial factor behind the rejection of novel food. However, ignorance may not always be the most vital barrier, and its effect as a barrier often increases with other factors. Moreover, how and what kind of information is communicated may affect the results. Indeed, many studies report differential effects of providing information on consumer acceptance. Both the message and the medium used for information provision impact consumer choice. In this sense, labels provide a powerful channel for communicating a product’s benefits, as they signal the product’s safety, health, and environmental or hedonic benefits [81,106].

Familiarity, disgust, food neophobia, and novel food technology neophobia have been among the most studied barriers to novel food consumption. Table 1 summarizes these barriers, their effects, and several related strategies suggested to overcome their adverse effects.

## 5. Conclusions

This review aimed to understand the consumer response to novel food by exploring the key barriers and drivers related to the behavioral side of novel food acceptance. Several factors, from personality traits to sustainability concerns, social context to disgust, play a role in the consumer response to novel foods, which is very complex and contextual. This review also revealed that studies often use different conceptual models and factors to understand consumer behavior in a product-oriented context, making it very difficult to understand and interpret the effects of behavioral barriers comparatively. Given the chaotic nature of the process, it is crucial to consider consumer expectations, potential barriers, and drivers in the early stages of novel food development. 

This review showed that despite the direct connection of biodiversity to agricultural productivity and other environmental challenges [2], the number of studies focusing on the relationship between the biodiversity-related benefits of introducing novel foods and their acceptance is very limited. 

Future research can aim to adopt a holistic approach to food behavior and understand how consumers perceive the value of diversity within their dietary patterns. Alternatively, research comparing the acceptance of different types of novel foods and their level of contribution to biodiversity conservation could provide valuable insights for policymakers and marketing strategists. Research on the longitudinal effects of interventions involving information provision and skills development on consumer acceptance of novel foods could also be precious. 

## 6. Practical Implications

The Prospect Theory explains that people tend to classify outcomes of a certain action as gains or losses and to evaluate those gains and losses relative to a subjective and context-dependent “reference point” [117]. When compared, consumers are more sensitive to losses than to gains, making them typically willing to take more risks when a decision involves potential losses. This loss-averse nature of consumers is related to “endowment bias” and “status quo bias”. Endowment bias refers to the tendency of people to be more sensitive about things they own [118]. Losses related to things people own are typically more painful than losses about things they do not own. Similarly, status quo bias explains that people tend to keep things the way they currently are, even when better alternatives are available [119]. The status quo or possessions are typically perceived as a reference point, and any deviation from this point is perceived as a loss or gain. Changing existing eating habits and incorporating novel foods into the diet is often perceived as a deviation that involves loss. 

As this review indicates, the motivations behind the intention to consume novel foods are often related to environmental sustainability, health, or animal welfare. The gains related to these motivations are frequently expected to occur in the future rather than immediately, and the outcomes are not always easy to discern. Unfortunately, people are typically myopic or short-sighted and value immediate gains compared to future ones. This tendency is called present bias [120]. As a result of present bias, people often settle for smaller gains because they occur over a shorter period. Among other factors, the impact of present bias can be observed in the tendency for even people who are highly motivated by the environmental benefits of novel foods to continue their current habits in favor of immediate convenience or pleasures.

Taken together, consumers’ intention to accept novel foods is undermined by their loss-averse nature and the influence of status quo, endowment, and present biases. The perceived loss of giving up their habits and the current situation is more worth the risk than the perceived potential gains in the future. Policies or marketing strategies can be designed using behavioral levers to encourage consumers to change their dietary habits in a direction that contributes to environmental benefits, such as preserving biodiversity. “Nudge” aims to design behavioral interventions that address biases for better social and individual outcomes. Nudges involve a set of interventions to smoothly encourage individuals toward a desired action by simply altering the environmental factors (i.e., cues) and providing information considering heuristics and cognitive biases associated with specific behaviors [121].

“Framing” is a potential tool for reshaping consumers’ perceptions of loss and gain. Different ways of presenting information elicit diverse emotional responses, influencing the selection of alternatives [122]. Consequently, behavior can be changed by altering whether consumers view an outcome as a gain or a loss. In the context of consumer acceptance of novel food, this strategy entails reframing the decision to forego current consumption, often perceived as a loss, as a gain. Conversely, it involves depicting the choice not to consume alternative novel foods, typically viewed as a gain, as a potential loss. For example, when providing information about underutilized varieties, emphasis is often placed on potential gains related to health, environment, climate change, traditions, and biodiversity. Using framing strategies, the focus of information provision can be shifted toward the relative potential losses if they are not consumed. Furthermore, framing underutilized crops as “forgotten products/varieties” may significantly impact consumer food purchasing decisions by reinforcing their perception of them as lost treasures. Complementarily, social norms are frequently employed to steer consumers toward desired behaviors [123]. “Injunctive social norms” can be leveraged to promote the purchase of novel foods made from underutilized crops. By placing pro-biodiversity labels on products, consumers receive a clear message regarding socially approved and expected behavior. 

## Figures and Tables

**Table 1:** Major barriers to novel food acceptance and related strategies.

Barrier	Explanation	Effect	Potential Strategies to Overcome Adverse Effects on Consumer Acceptance of Novel Food
Familiarity	A food is familiar if it is part of an individual’s diet [30]	Familiarity with a food develops certainty about expectations, and it often creates an advantage over unfamiliar or novel food products [21,30]. Not always enough for food acceptance [31].	Disguising novel food as an ingredient in familiar foods can increase acceptance [31,32,43,67,71,74,77,79,82,88,107,108]. Appropriate product design [31,43]. Communication of the sustainability benefits or production methods through labels or written messages [71,81,109]. Developing slightly different forms of known foods or a relation to known brands [18].
Disgust and food neophobia	Disgust is a strong feeling of dislike of a food. It is usually shaped by beliefs about the nature or origin of the food [80]. Food neophobia is defined as the reluctance to eat and/or avoidance of novel foods [35].	Disgust and food neophobia often co-exist and create a psychological barrier to the acceptance of novel food [26,68,74,78,80,86,88]. Their effect can suppress other factors that could positively affect novel food acceptance [80,110]. As the physical disadvantages of individuals increase, the effect of food neophobia may escalate [53]. Generally, there is a lower or no effect for new plant-based protein sources [51,111].	Positive sensory experience [78,80]. Disguising novel food as an ingredient in familiar foods [32,43,69,74,77,82,88,107,108,112]. Processed products (when the visibility of insects is a barrier) [86]. Providing information about environmental benefits [49,71]; environmental and health benefits [77,78]; nutritional health claims (on the label) [81]; the production of cultured meat in technical terms (countereffect) [49].
Novel Food Technology Neophobia (NFTN)	The fear of novel food technologies [30]	NFTN creates a psychological barrier to the acceptance of novel food. It often coexists with different concerns related to different technologies. Examples include perceived lack of naturalness [57,79] and safety concerns [113].	Increasing trust in science and certain technologies [56,58,79,114,115]. Building trust in science (3D-printed foods) [79]. Communicating personal benefits (3D-printed foods) [79]. Providing information about health and other personal benefits (3D-printed food) (no effect) [116].

## Data Availability

No new data were created or analyzed in this study. Data sharing is not applicable to this article.

## Appendix A

**Table A1:** Main attributes of novel foods.

Novel Food Attribute	Product	Study Region	Reference
Alternative protein	Microalgae breadsticks	Spain	[124]
Alternative protein	Microalgae	Germany	[115]
Alternative protein	Insect-based food products	The Netherlands	[81]
Alternative protein	Mealworm products such as Dutch beef stew (savory, Western), curry (savory, Asian), brownie (sweet, Western), and Indonesian spice cake (sweet, Asian)	The Netherlands	[31]
Alternative protein	Chocolate chip cookie with an ingredient from edible insects (10% of cricket flour)	Italy	[77]
Alternative protein	Surimi-based products shaped as pasta	Sweden	[70]
Alternative protein	Cakes, cookies, and waffles with insect ingredients (BSF LF)	Belgium	[43]
Alternative protein	Vexo (a meat alternative containing insect and plant protein)	The UK	[72]
Alternative protein	Cookie made with mealworm (*Tenebrio molitor*) flour	Brazil	[74]
Alternative protein	Chocolate bar with insect flour (*A. Domesticus*), whole crickets (*A. Domesticus*), chips containing insect flour (*A. Domesticus*), caramel worms (*T. Molitor*)	Italy	[69]
Alternative protein	Insect-based products	Serbia	[66]
Alternative protein	Plant-based and cultured meat	China	[114]
Alternative protein	Insect-based convenience foods	The Netherlands	[94]
Alternative protein	Whole edible insects and foods containing edible insects	The Netherlands	[85]
Alternative protein	Whole and processed mealworms (*Tenebrio molitor*)	Belgium, China, Italy, Mexico, the USA	[125]
Alternative protein	Meat, fish, eggs and milk obtained from animals raised with insect-based feed; protein food supplements based on insect flour (e.g., cricket flour); cookies made from wheat and insect flour; cookies containing visible insects	Italy	[44]
Alternative protein	Bread made with insect flour	Italy	[84]
Alternative protein	Insects and insect meal-reared beef, pork, poultry, fish	Italy	[83]
Alternative protein	Mealworms, grasshoppers, ants and insect-reared fish and pig	Denmark, Italy	[26]
Alternative protein	Edible insects	Norway, Portugal	[78]
Alternative protein	Edible insects	The USA	[126]
Alternative protein	Edible insects and insect-based products, such as cricket flour or edible insect-filled chocolate bars	Australia	[80]
Alternative protein	Edible insects	Australia	[82]
Alternative protein	Edible seaweed	Italy	[75]
Alternative protein	Insects	Spain	[90]
Alternative protein	Edible insects	Brazil	[68]
Alternative protein	A cookie, a muffin, date ball (none of them contain insect-based ingredients)	Poland	[46]
Alternative protein	Insects: black field cricket nymph, huhu beetle grub, mānuka beetle adult, porina caterpillar, locust nymph and wax moth larvae	New Zealand	[87]
Alternative protein	Insect-farmed fish	Italy	[127]
Alternative protein	Falafel with mealworm flour	England	[71]
Alternative protein	Whole and processed insect-based products	Germany	[86]
Alternative protein	Insect-based convenience foods	The Netherlands	[100]
Alternative protein	Insect-based convenience foods	The Netherlands	[103]
Alternative protein	Plant-based meat alternatives	China	[89]
Alternative protein	Meat analogues	China	[128]
Alternative protein, cellular agriculture	Cultured meat	USA	[102]
Alternative protein, cellular agriculture	Lab-grown meat	The USA, Singapore	[104]
Alternative protein, cellular agriculture	Cultured ground meat	The USA	[73]
Alternative protein, cellular agriculture	Cultured meat	China, India, Colombia, Switzerland	[98]
Alternative protein, cellular agriculture	Laboratory-grown meat	Brazil	[97]
Alternative protein, cellular agriculture	Cultivated meat	Singapore	[129]
Alternative protein, cellular agriculture	Cultured meat	Italy	[45]
Alternative protein, cellular agriculture	Cultured meat	Australia, China, England, France, Germany, Mexico, South Africa, Spain, Sweden, the USA	[48]
Alternative protein, cellular agriculture	Cultured meat	Italy	[47]
Alternative protein, cellular agriculture	Cultured meat	Singapore	[99]
Alternative protein, cellular agriculture	Artificial meat	China	[130]
Alternative protein, cellular agriculture	Laboratory-grown meat, plant-based meat	EU, the UK, the USA	[113]
Alternative protein, cellular agriculture	Cultured meat	Switzerland	[49]
Alternative protein, cellular agriculture	Precision fermentation (PF)-made egg products	Germany, Singapore, the USA	[50]
Alternative protein, underutilized	Pasta filled with spirulina, maki-sushi filled with spirulina, and spirulina jerky	Germany, France, The Netherlands	[32]
Alternative protein, cellular agriculture, 3D-printed foods	Insect-based foods, cultured meats, plant-based meat alternatives, and 3D-printed foods	Japan	[105]
Alternative protein, 3D-printed food	3D-printed sugar confectionary, carrots, a meal made from chicken and vegetable purees, pizza, pasta, and chocolate, snack made with insect flour	Australia	[57]
3D-printed food	3D-printed milk chocolate swirls, gummy candy carrots, and baked potato Smiles	Canada	[116]
3D-printed food	Not specified	Ireland	[79]
Biofortified crops	Pasta from yellow cassava and leafy vegetables	Nigeria	[51]
Functional food	High-protein egg-based recipes and single-use herb/spice packets on egg and protein intakes	The UK	[53]
Functional food	Fortified staple food (rice and bread)	Singapore	[52]
Functional food	Milk	Various European Countries	[131]
Gene editing, genetic modification, edible coating	Sliced apple product	Canada	[132]
Gene-edited and genetically modified foods	Soybean oil	South Korea	[133]
Gene-edited plant and animal products	Gene-edited rice and pork products	China	[58]
Genetically modified (GM) plant and animal products	GM soybean oil, GM-fed and GM salmon	Norway	[92]
Genetically modified (GM) animal products	GM pork product	The USA, China, Italy	[95]
Healthier meat	Healthier meat alternatives	Denmark, Spain, the UK	[67]
Newly developed food products	Not specified	Romania	[18]
Novel food technologies	Various	Australia, India, Singapore, the USA	[62]
Novel food technologies	Various	Ireland	[91]
Pulsed electric fields (PEF)	Not specified	European context	[134]
Seaweed products	Seaweed snack, salad, kelp noodles	The USA	[101]
Subtropical vegetables as novel crops	Not specified	South Korea	[76]
Technologies designed to control foodborne bacteria	Not specified	China, New Zealand	[64]
Underutilized food	Processed baobab (*Adansonia digitata* L.)	Malawi	[59]
Underutilized food	Breadfruit	Hawaii, the USA	[60]
Value-added surplus products (VASPs)	Soup, juice, granola bars, and pasta sauce	The USA	[106]
Value-added surplus products (VASPs)	Vegetable powder, vegetable snack, fermented product based on vegetables	Australia, The UK	[54]
Value-added surplus products (VASPs)	Upcycled ingredients of olive leaves	Italy	[55]
Value-added surplus products (VASPs), functional food	Yogurt that contains dietary fibers, natural flavorings with fruity aromas (banana flavor), and beneficial microbial cultures derived from halloumi whey and enriched with by-products (e.g., peel) of banana processing	Greece, Cyprus	[96]
Value-added surplus products (VASPs), functional food	Yogurt that contains dietary fibers, natural flavorings with fruity aromas (banana flavor), and beneficial microbial cultures derived from halloumi whey and enriched with by-products (e.g., peel) of banana processing	Greece, Cyprus, Uganda	[56]
Various novel food technologies	Vegetables (tomato as a concept stimuli)	New Zealand	[61]
Indoor agriculture	Leafy vegetables	Russia	[65]
Indoor agriculture	Leafy greens	The USA	[63]
Not specified	Various novel food	The USA, China, New Zealand	[93]
